# Prevalence of Gastroesophageal Reflux Disease and Associated Risk Factors in the Eastern Region, Saudi Arabia

**DOI:** 10.7759/cureus.19599

**Published:** 2021-11-15

**Authors:** Hussain A Al Ghadeer, Zahra E Alabbad, Salwa B AlShaikh, Shaheen U Ahmed, Ali A Bu-khamseen, Ali T Alhashem, Alaa H Alhamrani, Mohammed R AlGhadeer, Dhiyaa A Alibrahim, Bassil M Alkishi

**Affiliations:** 1 Paediatrics, Maternity and Children Hospital, AlAhsa, SAU; 2 Internal Medicine, King Faisal University, AlAhsa, SAU; 3 Internal Medicine, Almoosa Specialist Hospital, AlAhsa, SAU; 4 Internal Medicine, Dammam Medical Complex, Dammam, SAU; 5 Internal Medicine, King Fahad General Hospital, AlAhsa, SAU; 6 Family and Community Medicine, Al-Ahsa Family Medicine Academy, AlAhsa, SAU

**Keywords:** saudi arabia, prevalence, risk factors, gerdq, gastroesophageal reflux

## Abstract

Background

Gastroesophageal reflux disease (GERD) is a common upper gastrointestinal disorder characterized by heartburn and acid regurgitation. A higher incidence is found in Arab countries. Untreated GERD has a negative impact on individuals that interfere with daily activities and impaired quality of life. This study aims to estimate the prevalence of GERD and associated risk factors in the Eastern region, Saudi Arabia.

Material & Methodology

A descriptive cross-sectional study was carried out among 1517 healthy participants from the Eastern province of Saudi Arabia from May to August 2021. The sample was randomly collected through a structured self-administered questionnaire. The questionnaire was composed of questions related to sociodemographic and lifestyle characteristics as risk factors for GERD. The existence of GERD was assessed by using GERD Questionnaire (GerdQ) for diagnosing GERD, when the score is 8 or more.

Results

A total of 1517 participants were included in the study: 58.8% male, 41.2% female; 9% of whom were pregnant. The age of participants ranged from 18 to 58 with a mean age of 27.5 ± 11.4 years old. The existence of GERD was 20.6% among the total participants, in which their GerdQ scores were 3-7 (68.9%), 8-10 (22.1%), and 8-11 (8.5%). The higher risk groups of having GERD were pregnant women, smoker, being male, regular usage of analgesia, soft drinks, and having a family history of GERD.

Conclusion

This study showed the prevalence of GERD among the general population of the Eastern region, Saudi Arabia was 20.6%. Several sociodemographic and lifestyle characteristics were associated with the disease. Further studies are needed to explore the role of psychological factors in developing GERD.

## Introduction

One of the normal physiologies of the gastrointestinal tract is a backflow of gastric contents from the stomach to the esophagus. When this process causes burning feeling in the retrosternal area due to an injury on esophagus mucosa at least once per week this is called gastroesophageal reflux disease (GERD) [1]. The heartburn can radiate to the neck and it is aggressive after meals or during lying down position [2]. GERD has also extra-esophageal symptoms like chronic cough, asthma, and sometimes tissue lesions [3]. GERD has unknown causes, it is mostly caused by multifactorial pathogenesis like esophagogastric junction dysfunction, hypersensitivity of the esophagus, impaired esophageal bolus transit, and high intragastric pressure [4]. There are risk factors that increase the chance of getting GERD like age, obesity, lifestyle, taking non-steroidal anti-inflammatory drugs (NSAIDs), and smoking [5]. GERD is usually treated by proton pump inhibitors (PPIs) and lifestyle modification [6,7,8]. Depending on a recent systematic review for GERD management, one of the most important management is lifestyle modification like decreasing weight, smoking cessation, and avoid heavy meals at night [4]. If GERD is left without treatment, it will lead to dangerous complications like Barrett’s esophagus then esophagus cancer [1,5].

Recent epidemiological studies showed there was increasing GERD prevalence worldwide [3]. The worldwide estimated prevalence of GERD ranged from 15 to 25%, Saudi Arabia reported 15-45.4%, western Asia reported 10-20%, the Middle East reported 8.7-33.1%, and eastern Asia reported less than 10% [1,5]. Western countries and the USA have GERD prevalence higher than Asia, it ranges from 10 to 30% and more [9]. Such a highly prevalent condition that affects the gastrointestinal tract also has an impact on patient’s health-related quality of life (HRQL) [10].

Quality of life is represented by some components like degree of satisfaction, living conditions, accomplishments, functionality, cultural contexts, and finally, spirituality. In the field of health, ethical considerations play a major role in the individual’s life regarding multiple aspects, including doing what is correct in terms of respect, dignity, principles, and moral values. In the context of providing healthcare services, offering appropriate therapeutic choices by professionals is a good way to guarantee a better quality of life [11].

## Materials and methods

Aim

This study aims to estimate the prevalence and associated risk factors of GERD among the general population in the Eastern region, Saudi Arabia.

Study design and participants

A population-based cross-sectional study was carried out from May to August 2021 to investigate the prevalence and risk factors of GERD in the Eastern region, Saudi Arabia. The study included all participants who were aged more than 18 years old and healthy. The exclusion criteria were any participant who is: 1) less than 18 years old; 2) on regular usage of PPI or H_2_ blocker; 3) having gastrointestinal diseases; 4) underwent abdominal surgery; and 5) having organ insufficiency. The minimal required sample size was 385 by using a confidence interval (CI) level of 95%, a standard deviation of 0.5, and a margin of error of 5%. The sample size of this study was 1517 achieved through a structured self-administrated questionnaire that was distributed online through social media.

Data collection instrument and procedures

The data was collected through a structured questionnaire that was distributed online and composed of three sections. The first section covered the sociodemographic data including age, gender, family income, and level of education. It also included questions related to pregnancy. The second section covered the risk factors and lifestyle. The last section was for screening and diagnosis of GERD. A reliable and validated GerdQ was used. This GerdQ consisted of six items used as a diagnostic tool for GERD.

GerdQ Scoring

GerdQ is used as a diagnostic tool or diagnosis of GERD. This questionnaire is composed of six items in which four items are considered as positive predictors (heartburn, regurgitation, sleep disturbance due to heartburn and/or regurgitation, and use of over-the-counter medication other than that used for GERD). The remaining two items were considered as negative predictors (epigastric pain and nausea). Likert scale was used for scoring as 0= non, 1 = 1 day, 2 = 2-3 days, and 3 = 4-7 days with reversed scoring rate for negative predictors items (3= non). The cut-off of diagnosis of this tool is more than 8. The specificity and sensitivity of this GerdQ are 71% and 65%, respectively [12].

Data Analysis

After data was extracted, it was revised, coded, and fed to statistical software IBM SPSS version 22 (SPSS, Inc. Chicago, IL). All statistical analysis was done using two-tailed tests. P-value less than 0.05 was statistically significant. For the GERD scale, the total score was calculated, and a diagnosis of GERD was made using a cut-off score of >8 [12]. As for the severity of GERD, score 0-2 points were considered as < 50% likelihood of GERD, 3-7; 50% likelihood of GERD, 8-10; 79% likelihood of GERD, and 11-18; 89% likelihood of GERD. Descriptive analysis based on the frequency and percent distribution was done for all variables including participants’ data, pregnancy, and family history of GERD. Also, participants’ risk factors and lifestyles were displayed all to GERD. Crosstabulation was used to assess the distribution of GERD according to parents’ data and risk factors with lifestyle. Relations were tested using the Pearson chi-square test. To identify the most significant predictors for having GERD, a multiple logistic regression model was applied to detect the adjusted odds ratio.

## Results

The study included 1517 participants who fulfilled the inclusion criteria and completed the study questionnaire. Participants’ ages ranged from 18 to 58 with a mean age of 27.5 ± 11.4 years old. Exact of 58.8% were males, 68.7% were married, and 9% of married females were pregnant; 35.3% were in their 3rd trimester while 25.5% were in 1st trimester. As for educational level, 73.8% were university graduated and 42.3% were not employed, 39% were non-healthcare workers but 18.8% were healthcare workers.

**Figure 1:**
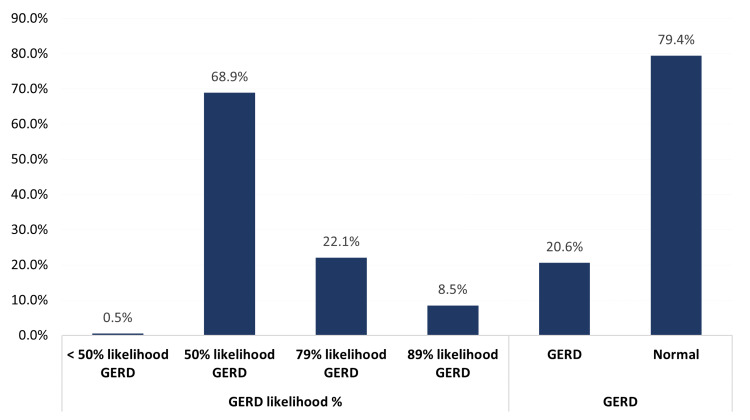
Prevalence of GERD among public in Eastern region, Saudi Arabia GERD: gastroesophageal reflux disease

Figure 1 shows the prevalence and severity of GERD among the public in the Eastern region, Saudi Arabia. Out of 1517, 312 (20.6%) (95% CI of 19% to 23%) of the participants were found to have GERD. According to the likelihood, GERD is classified into 50%, 79%, and 89% based on the total scores. Fifty percent of likelihood was 68.9%, 79% of likelihood was 22.1%, and 89% of likelihood was 8.5%.

Table 1 illustrates the distribution of GERD among study participants in Eastern Region, Saudi Arabia by their data. GERD was detected among 25.4% of the old age group (45 years or more) in comparison to 15.6% of young, aged participants with recorded statistical significance (P=0.002). Also, 26.1% of females complained of GERD compared to 16.7% of females (P=0.001). GERD was detected among 45.1% of pregnant females compared to 15.6% of non-pregnant groups (P=0.001). A higher percent of GERD was detected in the third trimester (66.7%) than 1st trimester (38.5%) with no statistical significance.

**Table 1:** Distribution of GERD among study participants of Eastern Region, Saudi Arabia by their personal data P: Pearson X^2^ test; GERD: gastroesophageal reflux disease *P < 0.05 (significant)

Personal data	Total		GERD				P-value
			GERD		Normal		
	No	%	No	%	No	%	
Age in years							0.002*
18-25	507	33.4%	79	15.6%	428	84.4%	
25-34	400	26.4%	82	20.5%	318	79.5%	
35-44	331	21.8%	80	24.2%	251	75.8%	
45+	279	18.4%	71	25.4%	208	74.6%	
Gender							0.001*
Male	625	41.2%	163	26.1%	462	73.9%	
Female	892	58.8%	149	16.7%	743	83.3%	
Marital status							0.112
Single	458	30.2%	83	18.1%	375	81.9%	
Married	1042	68.7%	223	21.4%	819	78.6%	
Divorced	17	1.1%	6	35.3%	11	64.7%	
Are you pregnant?							0.001*
Yes	51	9.0%	23	45.1%	28	54.9%	
No	514	91.0%	80	15.6%	434	84.4%	
Pregnancy duration							0.065
1st trimester	13	25.5%	5	38.5%	8	61.5%	
2nd trimester	20	39.2%	6	30.0%	14	70.0%	
3rd trimester	18	35.3%	12	66.7%	6	33.3%	
Educational level							0.648
Below secondary	37	2.4%	9	24.3%	28	75.7%	
Secondary	361	23.8%	69	19.1%	292	80.9%	
University/above	1119	73.8%	234	20.9%	885	79.1%	
Job title							0.235
Not employed	641	42.3%	125	19.5%	516	80.5%	
Non-healthcare worker/student	591	39.0%	118	20.0%	473	80.0%	
Healthcare worker/student	285	18.8%	69	24.2%	216	75.8%	

Table 2 reveals risk factors and lifestyle of GERD among public of Eastern region, Saudi Arabia. GERD was detected among 25.9% of participants who eat more than three meals per day compared to 18% of those who eat three meals with detected statistical significance (P=0.034). Also, 27.6% of participants who frequently had soft drinks complained of GERD compared to 20.8% who frequently had stimulants (coffee and tea), and 18.1% of those who frequently had citrus juice but only 16.7% of those who did not have any type had GERD (P=0.027). The exact 30.3% of smokers complained of GERD compared to 19% of non-smokers (P=0.001). Also, 34.8% of participants with a family history of GERD complained of the disease in comparison to 14% of those without (P=0.001). Other factors including physical activity, analgesics intake, eaten food were not significantly associated with GERD among study participants.

**Table 2:** Risk factors and lifestyle of GERD among public of Eastern Region, Saudi Arabia P: Pearson X^2^ test; GERD: gastroesophageal reflux disease *P < 0.05 (significant)

Risk factors and life style	Total		GERD				P-value
			GERD		Normal		
	No	%	No	%	No	%	
Physical activity > 30 minutes/week							0.639
Never	593	39.1%	131	22.1%	462	77.9%	
1 time/week	303	20.0%	62	20.5%	241	79.5%	
2-3 times/week	352	23.2%	69	19.6%	283	80.4%	
> 3 times/week	269	17.7%	50	18.6%	219	81.4%	
Most type of analgesics used							0.134
None	360	23.7%	67	18.6%	293	81.4%	
NSAID	107	7.1%	25	23.4%	82	76.6%	
Paracetamol	1019	67.2%	209	20.5%	810	79.5%	
Others	31	2.0%	11	35.5%	20	64.5%	
Number of daily meals							0.034*
< 3 meals	637	42.0%	143	22.4%	494	77.6%	
3 meals	745	49.1%	134	18.0%	611	82.0%	
> 3 meals	135	8.9%	35	25.9%	100	74.1%	
Types of eaten foods							0.362
Greasy/fatty	660	43.5%	149	22.6%	511	77.4%	
Spicy	274	18.1%	55	20.1%	219	79.9%	
Sugar (chocolate)	308	20.3%	58	18.8%	250	81.2%	
Healthy diet	275	18.1%	50	18.2%	225	81.8%	
Most type of drinks							0.027*
Stimulus (Tea, Coffee)	891	58.7%	185	20.8%	706	79.2%	
Soft drinks	192	12.7%	53	27.6%	139	72.4%	
Citrus juice	105	6.9%	19	18.1%	86	81.9%	
None	329	21.7%	55	16.7%	274	83.3%	
Smoker							0.001*
Yes	211	13.9%	64	30.3%	147	69.7%	
No	1306	86.1%	248	19.0%	1058	81.0%	
Family history of gastroesophageal disease							0.001*
Yes	477	31.4%	166	34.8%	311	65.2%	
No	1040	68.6%	146	14.0%	894	86.0%	
Salt or pickles consumption with meal							0.387
Yes	999	65.9%	199	19.9%	800	80.1%	
No	518	34.1%	113	21.8%	405	78.2%	
Having fast food							0.770
Yes	471	31.0%	99	21.0%	372	79.0%	
No	1046	69.0%	213	20.4%	833	79.6%	

Table 3 reveals multiple stepwise logistic regression models for predictors of GERD among the general population of Eastern region, Saudi Arabia. Among all factors, what is shown in the table were the most significant predictors for having GERD keeping all other factors constant. Pregnancy was associated with nearly three times more likely for GERD than non-pregnant (odds ratio [OR]=2.67), smokers had also nearly tripled the risk for GERD than non-smokers (OR=2.56), male gender was associated with a doubled risk for GERD (OR=2.21), regular use of analgesics recorded doubled risk for GERD (OR=2), and family history recorded 47% more likelihood for GERD which was 21% for old age and 10% for soft drinks intake.

**Table 3:** Multiple stepwise logistic regression model for predictors of GERD among general population, Eastern Region, Saudi Arabia ORA: adjusted odds ratio; CI: confidence interval; GERD: gastroesophageal reflux disease *P < 0.05 (significant)

Predictors	P-value	ORA	95% CI for OR	
			Lower	Upper
Old age (more than 45 years)	0.048*	1.12	1.0	1.27
Male	0.0011*	2.21	1.65	2.29
Pregnancy	0.0011*	2.67	1.94	3.67
Soft drinks	0.0021*	1.10	1.11	1.25
Smoking	0.0011*	2.56	2.72	4.67
Regular using of analgesics	0.001*	2.00	1.36	2.70
Family history of GERD	0.0381*	1.47	1.02	2.10

## Discussion

The current study was conducted to identify the prevalence of GERD patients in the Eastern region, Saudi Arabia and also to detect determinants of having GERD among the study population. GERD is a public disease with annoying symptoms and a significant influence on patients’ quality of life [13].

Recently, a systematic review showed that the prevalence of GERD is 18.1-27.8% in North America, 8.8-25.9% in Europe, 2.5-7.8% in East Asia, 8.7-33.1% in the Middle East, 11.6% in Australia, and 23.0% in South America [14]. The surge in GERD incidence has many explained and unexplained factors including older age, race, male sex, analgesics use, having some types of food and drinks, smoking, family history of GERD, obesity, and inadequate physical activity. Most of these factors are linked to a patient’s lifestyle [15].

The current study revealed that nearly one-fifth (20.6%; 95% CI: 19%-23%) of the participants complained of GERD which was of 50% likelihood among the vast majority of them (69%). This was higher than what was reported by Chen et al. [16] in China where the prevalence of heartburn and/or acid eructation occurring at least weekly was 6.2%. The point prevalence of GERD symptoms 2.3% (95% CI, 1.8%, 2.8%). Also, Cho et al. [17] in Korea assessed a lower incidence of GERD among the population. Their study revealed that the prevalence of GERD, defined as heartburn and/or acid regurgitation experienced at least weekly, was 3.5% (95% CI, 2.6-4.5). There were many other studies [18-21] that assessed the prevalence of GERD and reflux esophagitis among different populations. The prevalence of GERD and reflux esophagitis ranged from 1.4 to 52.1%. This wide range may be attributed to many factors such as population lifestyle, culture, the definition used for GERD, associated co-morbidities, methods for assessment of GERD, age categories, and dietary habits variations. Locally, Alsulobi et al. [22] found that 61.8% of the population in Arar City, Northern Saudi Arabia reported a loss of appetite as an associated condition, 57% reported nausea and vomiting, 55.9% indigestion, 55.4% food regurgitation, and 41.4% chest pain. Alsuwat et al. [1] estimated that the prevalence of GERD among the Saudi participants was 28.7% which is similar to the current study findings. A higher prevalence for GERD was detected in Riyadh and a lower incidence was estimated in was the Western region of Saudi Arabia [23,24].

As for risk factors, the multiple logistic regression model showed that smoking was the most significant predictor for having GERD. This was in concordance with many literature studies which assessed the role of smoking in developing gastritis and GERD [25-27]. Also, pregnant women had a highly significant effect on developing GERD that could be physiological. Other factors included male gender, regular use of analgesics, family history of GERD, old age, and frequent intake of soft drinks. All those detected were proved as a major risk factor by many previous studies [28, 29].

## Conclusions

In conclusion, the current study showed that about 20.6% of participants complained of GERD to be mainly of mild degree. Also, the prevalence of GERD in the study population is somewhat higher than in many other countries especially western countries and countries of East Asia. Smoking, analgesic use, male gender, old age, and family history were the most significant predictors for developing GERD. Further detailed assessment of risk factors especially lifestyle and dietary habits are required to minimize the disease frequency by avoiding modifiable risk factors. Also, lifestyle interventions in GERD including avoidance of foods that may precipitate reflux episodes and heartburn and behavioral changes with weight loss, smoking cessation, head of the bed elevation may play a significant role in improving clinical symptoms of the disease and quality of life.

Limitations

Recall bias is one of the limitations that is common in these types of studies. Second limitation is that the sample size should be larger for external validity. Furthermore, lifestyle factors should be more specific such as the interval between last meal and sleep, sleeping habits. The finding of this current study highlighted the high prevalence of GERD in this region and further studies are needed to validate these results and to find out the possible causes of such prevalence because GERD has an impact on well-being.
